# A regional trauma system to optimize the pre-hospital triage of trauma patients

**DOI:** 10.1186/s13054-015-0835-7

**Published:** 2015-03-18

**Authors:** Pierre Bouzat, François-Xavier Ageron, Julien Brun, Albrice Levrat, Marion Berthet, Elisabeth Rancurel, Jean-Marc Thouret, Frederic Thony, Catherine Arvieux, Jean-François Payen, for TRENAU group

**Affiliations:** Department of Anaesthesiology and Critical Care, Grenoble University Hospital, Hôpital Albert Michallon, BP 217, F-38043 Grenoble, France; Grenoble Alps University, F-38000 Grenoble, France; Emergency Medical Service, Annecy Hospital, F-7400 Annecy, France; Department of Critical Care, Annecy Hospital, F-74000 Annecy, France; Emergency Medical Service, Grenoble University Hospital, Hôpital Albert Michallon, BP 217, F-38043 Grenoble, France; Department of Critical Care, Chambery Hospital, F-73000 Chambery, France; Department of Medical Imaging, Grenoble University Hospital, Hôpital Albert Michallon, BP 217, F-38043 Grenoble, France; Department of Visceral Surgery, Grenoble University Hospital, Hôpital Albert Michallon, BP 217, F-38043 Grenoble, France

## Abstract

**Introduction:**

Pre-hospital triage is a key element in a trauma system that aims to admit patients to the most suitable trauma center, and may decrease intra-hospital mortality. We evaluated the performance of a pre-hospital procedure in a regional trauma system through measurements of the quality of pre-hospital medical assessment and the efficacy of a triage protocol.

**Methods:**

Our regional trauma system included 13 hospitals categorized as Level I, II or III trauma centers according to their technical facilities. Each patient was graded A, B or C by an emergency physician, according to the seriousness of their injuries at presentation on scene. The triage was performed according to this grading and the categorization of centers. This study is a registry analysis of a three-year period (2009 to 2011).

**Results:**

Of the 3,428 studied patients, 2,572 were graded using the pre-hospital grading system (Graded group). The pre-hospital gradation was closely related with injury severity score (ISS) and intra-hospital mortality rate. The triage protocol had a sensitivity of 92% (95% confidence interval (CI) 90% to 93%) and a specificity of 41% (95% CI 39% to 44%) to predict adequate admission of patients with ISS more than 15. A total of 856 patients were not graded at the scene (Non-graded group). Undertriage rate was significantly reduced in the Graded group compared with the Non-graded group, with a relative risk of 0.47 (95% CI 0.40 to 0.56) according to the definition of the American College of Surgeons Committee on Trauma (*P* <0.001). Where adjusted for trauma severity, the expected mortality rate at discharge from hospital was higher than observed mortality, with a difference of +2.0% (95% CI 1.4 to 2.6%; *P* <0.01).

**Conclusions:**

Implementation of a regional trauma system with a pre-hospital triage procedure was effective in detecting severe trauma patients and in lowering the rate of pre-hospital undertriage. A beneficial effect on outcome of such an organization is suggested.

**Electronic supplementary material:**

The online version of this article (doi:10.1186/s13054-015-0835-7) contains supplementary material, which is available to authorized users.

## Introduction

Severe trauma remains a major issue for public healthcare worldwide. Approximately 5.8 million people die annually from traumatic injuries, representing 10% of deaths worldwide [1]. The optimization of trauma care is based on improving the medical decisions taken to provide appropriate treatments. Achieving this goal is possible with an organized trauma system that allocates appropriate resources of care according to the initial condition of each trauma patient. Implementation of such a trauma system was shown to decrease mortality and severe disability of patients admitted in a Level-I trauma center [2]. Thus, pre-hospital triage is a key element in a trauma system that aims to admit patients with severe trauma to the most suitable trauma center. Indeed, it may decrease intra-hospital mortality and avoid secondary time-consuming inter-hospital transfer [3]. Field triage schemes have been established by the American College of Surgeons Committee on Trauma (ACSCOT) to ensure correct admission of trauma patients to designated trauma centers in the USA [4].

**Figure 1:**
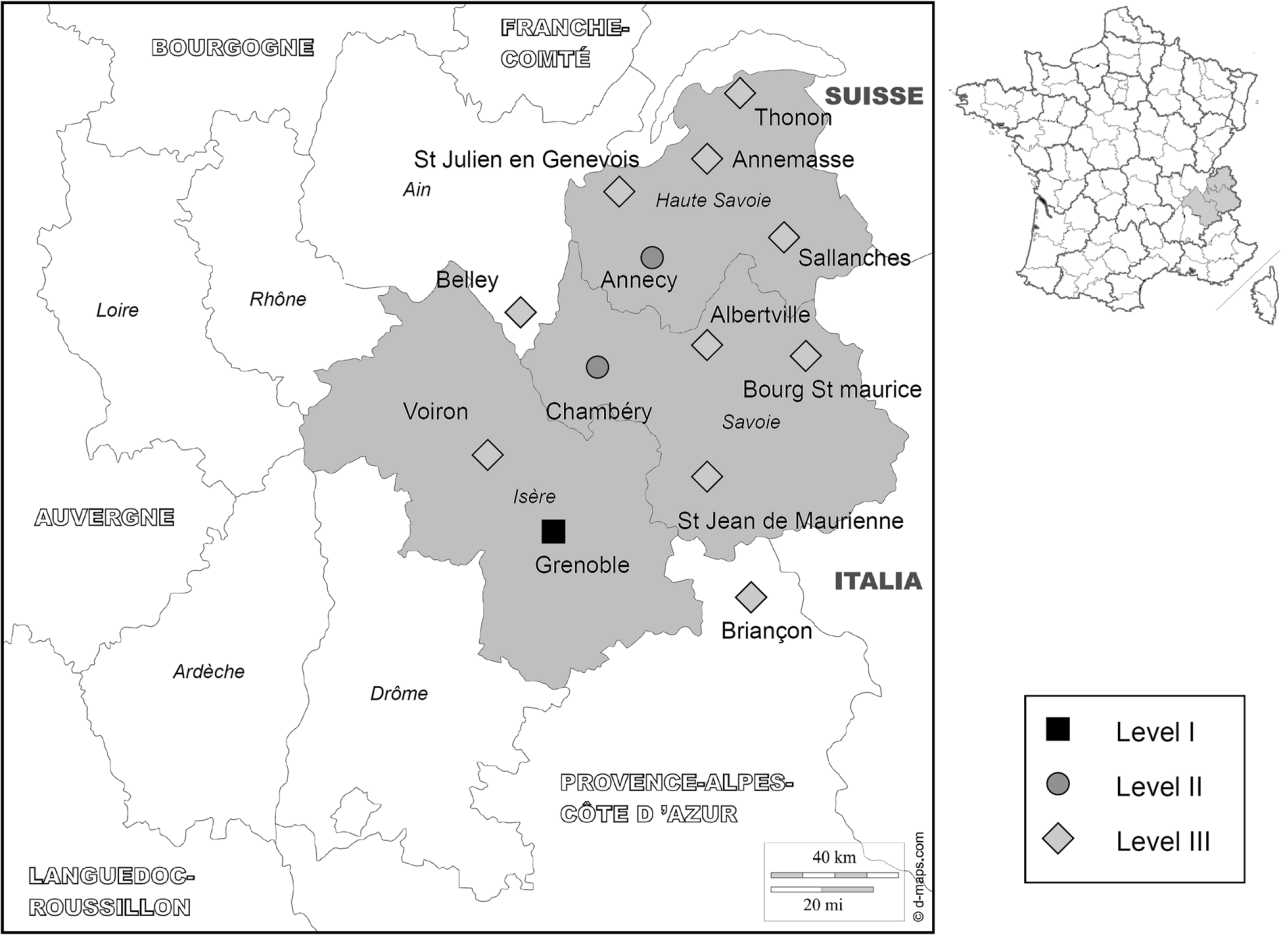
**Map of the trauma system of the Northern French Alps (TRENAU).** TRENAU is located in the Rhône-Alpes Region with three subareas: Isère, Savoie and Haute-Savoie (in grey). The trauma centers were designated according to their technical facilities, from Level-I to Level-III.

Although there is a growing interest in developing regional trauma systems across European countries [5], the reality of their implementation and impact is unknown. The Northern French Alps Trauma System (TRENAU), created in 2008 by Grenoble university hospital [6], accounts for more than two million inhabitants across a mountainous area with high seasonal variability. It is based upon matching the resources of each hospital participating to the network and the categorization of trauma severity at the scene. The TRENAU federates 22 hospitals within a regional area (Figure 1), of which 13 are designated as Level I, II or III trauma centers depending on their technical facilities (see Additional file 1). The TRENAU aims at directing the most severe patients to a Level I or Level II center by an adequate on-scene triage. Many level-III centers have also been designated to face the high seasonal attendance at mountain sport resorts. The emergency medical service (EMS) is organized according to a two-tiered activation system in France. A basic life support fire department ambulance is the first level. The second level includes an advanced life support physician-staffed ambulance that is activated for patients with severe trauma according to the French Vittel criteria (see Additional file 1). In this situation, each patient is graded A, B or C by an emergency physician, according to the seriousness of their injuries at presentation on scene. The patient is then referred to the most appropriate trauma center. We hypothesized that this organization would optimize the triage of trauma patients in allocating appropriate resources of care according to the on-scene medical assessment. The aim of the present study was to evaluate: (1) the quality of the pre-hospital grading protocol to detect the most severe trauma patients and (2) the accuracy of the TRENAU procedure to perform an adequate triage. Since the pre-hospital triage procedure might not always be applied to all trauma patients, we compared the rates of undertriage and overtriage between two groups of patients, those who were graded and those not.

## Materials and methods

The TRENAU contains one Level-I trauma center, two Level-II centers and ten Level-III centers. A registry of the TRENAU was developed in 2009 to collect data prospectively according to the Utstein-style [7]. Raw data were prospectively collected on paper by the in-charge physicians and were entered monthly into an electronic database. Research technicians provided continuous monitoring of the completeness and correctness of the database, and collected patient outcome at hospital discharge. This registry has since collected medical data relating to the whole process of trauma management, from the scene to admission in the intensive care unit. The Regional Institutional Ethics Committee approved the implementation of the TRENAU registry (*Comité d’Ethique des Centers d’Investigation Clinique de l’inter-région Rhône-Alpes-Auvergne*, IRB number 5708) and, given its observational nature, waived the requirements for written informed consent from each patient.

**Figure 2:**
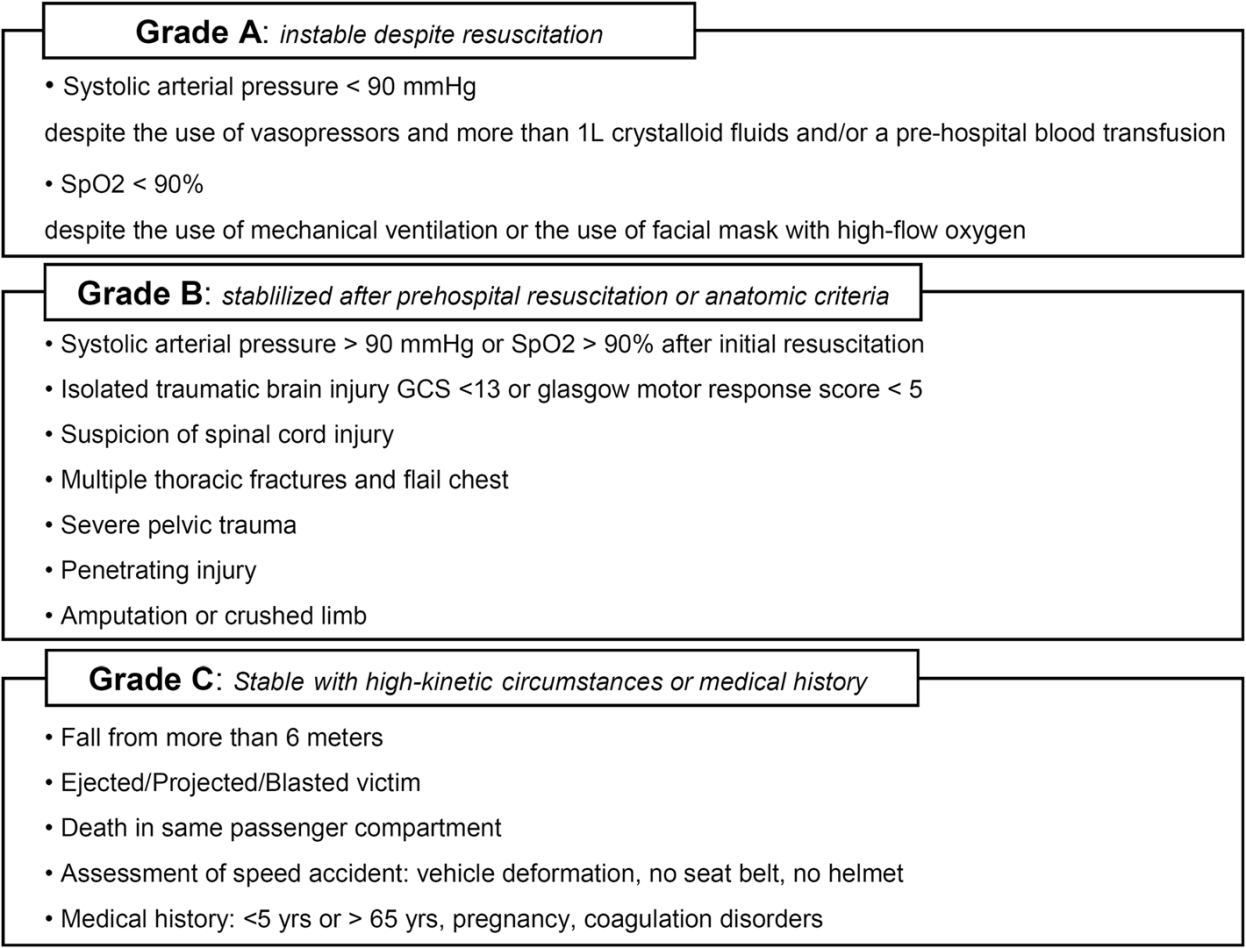
**Grading system for on-scene evaluation of trauma victims, according to the French Vittel triage criteria [** 8 **,** 9 **].** Hemodynamic instability is defined as a systolic arterial blood pressure of less than 90 mmHg despite the use of vasopressors and more than 1 L crystalloid fluids and/or a pre-hospital blood transfusion. Respiratory instability is defined as a SpO2 < 90% despite the use of mechanical ventilation and/or the use of a face mask with high-flow oxygen. GCS: Glasgow coma scale; SpO2, pulse oxygen saturation.

**Figure 3:**
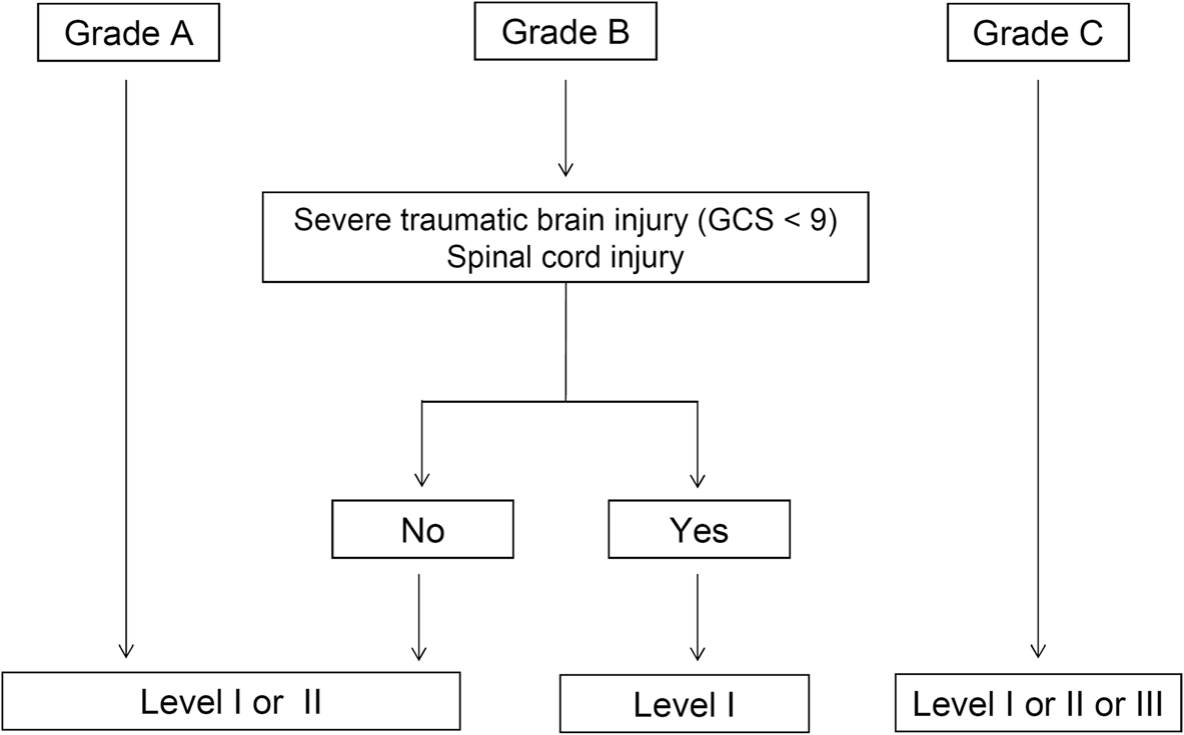
**Initial orientation according to grading tool scale and technical facilities of trauma centers affiliated with the Trauma System of the Northern French Alps (TRENAU).** GCS: Glasgow coma scale.

All studied patients in the registry over a three-year period (2009 to 2011) were included if severe trauma was suspected in the pre-hospital setting using the French Vittel triage criteria [8,9]. Trauma patients with cardiac arrest at the scene or those initially admitted to a center outside of the TRENAU were excluded from the analysis. The triage procedure integrated the on-scene assessment of the trauma severity by an emergency physician, which is referred to one EMS dispatch center for regulation before referral to the nearest and most suitable trauma center. The grading system uses criteria based on physiological findings, anatomical regions affected and mechanisms of injury, as described by the field triage decision scheme of the ACSCOT [10]. Additionally, the TRENAU grading system incorporates the responses to treatment during the pre-hospital resuscitation. Each patient was thus graded as one of three levels of clinical severity, that is, A, B or C, adapted from the French Vittel triage criteria (Figure 2). This categorization permitted the allocation of each patient to the most suitable trauma center according to the TRENAU algorithm (Figure 3). Patients may not benefit from the triage procedure because of medical team unavailability, low compliance with the procedure, or a false assessment of the initial severity. Therefore, we defined two different groups of patients during the study period: graded group for patients categorized by the pre-hospital triage protocol or non-graded group for patients with no pre-hospital medical assessment.

We first evaluated the quality of the pre-hospital grading system through its ability to detect the most severe trauma patients as defined by an injury severity score (ISS) of more than 15 [11]. ISS was calculated using the abbreviated injury score (AIS) 2005 catalogue. The accuracy of the pre-hospital triage protocol was also studied in these patients. An adequate triage was defined by two situations: (1) a trauma patient with ISS more than 15 admitted directly to a Level-I or Level-II trauma center (ACSCOT definition [10]) and (2) a trauma patient with ISS more than 15 admitted to a Level-I or Level-II trauma center or admitted to a Level-III trauma center if treated appropriately without inter-hospital transfer (TRENAU definition).

We also compared rates of undertriage and overtriage between the graded and the non-graded groups. According to the ACSCOT definition, undertriage describes the admission of trauma patients with an ISS of more than 15 to a Level-III trauma center. Overtriage is defined as the admission of trauma patients with ISS of 15 or less to a Level-I or Level-II trauma center [10]. We also used other definitions of undertriage and overtriage in order to take into account our rural environment (TRENAU definition). In this context, undertriage was considered in two other situations: 1) a patient with ISS of more than 15 admitted initially to a Level-III trauma center before being transferred to a Level-I or Level-II trauma center. This secondary transfer did not include technical stops in a Level-III trauma center, as defined by an optimization of hemodynamics and/or ventilation before inter-hospital transfer; and 2) a patient with an ISS of more than 15 who died from trauma in a Level-III trauma center. We also defined overtriage as the admission of patients with ISS of 15 or less to an emergency room, generating the activation of a trauma team in a Level-I or Level-II trauma center.

Descriptive statistics included frequencies and percentages for categorical variables, and the mean (SD or 95% confidence interval, CI) for continuous variables. The probability of survival at discharge from hospital was calculated using the Trauma score – Injury Severity Score (TRISS) methodology [12,13]. The *W* statistic was used to determine the difference between observed survivors and expected survivors from the TRISS model predictions [14]. Comparisons used Student’s t test or the Mann–Whitney test where appropriate for continuous variables, and Fisher’s exact test for categorical variables (Stata 12.0; Stata Corp, College Station, TX, USA). A *P* value of 0.05 or less was considered statistically significant.

## Results

Of the 3,689 patients recorded in the TRENAU registry during the study period, 261 were excluded from the analysis due to on-scene cardiac arrest (115 patients), initial management by a medical team not participating in the TRENAU (46 patients), and missing important data impeding the measurements of triage (100 patients). A cohort of 3,428 patients was thus retained for the analysis. Of these, 2,552 patients were referred to Level-I or Level-II trauma centers, and 876 patients were admitted to Level-III centers.

Road traffic and mountain accidents were leading causes of injury in the study population (Table 1). An ISS score of more than 15 was found in 48% of patients. There were 2,572 patients (75% of the population) who were graded using the pre-hospital grading system (Graded group). In this group, Type-A, Type-B and Type-C severity levels were found in 7%, 24% and 44% of the total population, respectively. The pre-hospital grading procedure could accurately detect patients with an ISS more than 15: 89% (95% CI 85% to 93%) in Type-A patients, 64% (95% CI 61% to 68%) in Type-B patients, and 31% (95% CI 28% to 33%) in Type-C patients (*P* <0.01). Similar findings were obtained regarding mortality rates at hospital discharge (*P* <0.01): 40% (95% CI 34% to 47%), 8% (95% CI 6% to 10%), and 1% (95% CI 0% to 1%) in Type-A, Type-B and Type-C patients, respectively (*P* <0.01). Using the TRENAU definition, the sensitivity and specificity of the triage protocol to predict adequate admission of patients with severe trauma were 92% (95% confidence interval 90% to 93%) and 41% (95% CI 39% to 44%), respectively (Table 2).

**Table 1:** **Characteristics of the 3,428 severe trauma patients**

**Variable**	**Value**
Included patients per year, number (%):	
2009	1070 (31)
2010	1163 (34)
2011	1195 (35)
Age, years (mean, SD)	37 ± 19
Sex male, number (%)	2,612 (76)
Mechanism of injury, number (%):	
Road traffic accidents	1495 (44)
Falls	652 (19)
Skiing accidents	533 (16)
Other mountain accident	337 (10)
Penetrating injuries	275 (8)
Others	114 (3)
Helicopter transport, number (%)	1264 (37)
Initial GCS score, number (%):	
3 to 8	394 (12)
9 to 13	314 (9)
14 to 15	2641 (79)
Initial SBP <90 mmHg, number (%)	204 (6)
Initial assessment of SpO2 < 90%, number (%)	248 (8)
ISS, number (%):	
<16	1762 (52)
16 to 24	756 (22)
25 to 34	600 (18)
>34	272 (8)
Overall AIS score ≥3, number (%)	2406 (70)
Head AIS ≥3, number (%)	826 (24)
Chest AIS ≥3, number (%)	1107 (32)
Abdomen AIS ≥3, number (%)	381 (11)
Pelvic AIS ≥3, number (%)	241 (7)
Limbs AIS ≥3, number (%)	640 (19)
Spinal cord injury, number (%)	125 (4)

AIS, abbreviated injury score; GCS, Glasgow Coma Scale; ISS, injury severity score; SBP, systolic blood pressure; SD, standard deviation; SpO2, pulse oxygen saturation.

**Table 2:** **Performance of the pre-hospital medical assessment in the Graded group using ACSCOT and TRENAU definition**

	**Number**	**ISS more than 15**	**Sensitivity**	**Specificity**	**PPV**	**NPV**
		**number (%)**	**% (95% CI)**	**% (95% CI)**	**% (95% CI)**	**% (95% CI)**
ACSCOT^a^	2572	1191 (46)	83 (80 to 85)	23 (21 to 26)	48 (46 to 51)	61 (56 to 65)
TRENAU^b^	2572	1191 (46)	92 (90 to 93)	41 (39 to 44)	58 (55 to 60)	85 (82 to 87)

^a^Adequate triage of the ACSCOT: a trauma patient with ISS more than 15 admitted to a Level I or II trauma center; ^b^adequate triage of the TRENAU: a trauma patient with ISS more than 15 admitted to a Level I or II trauma center, or admitted to a Level-III without secondary transfer to a Level I or II. CI: confidence interval; ISS, injury severity score; PPV: positive predictive value; NPV: negative predictive value.

There were 856 patients (25% of the population) who were not graded on the scene (Non-graded group). The reasons for the absence of grading were the absence of pre-hospital assessment by a physician (n = 453 patients) or the non-use of the grading scale by the pre-hospital emergency physician (n = 403 patients). Patients in this group were more likely to have had a skiing accident and have a higher ISS score compared to the Graded group (Table 3). Undertriage rates were significantly reduced in the Graded group compared to the Non-graded group whatever the definition used for appropriate triage, that is, ACSCOT or TRENAU (Table 4). The grading systems significantly decreased the relative risk of undertriage by 0.47 (95% CI 0.40 to 0.56) and 0.33 (95% CI 0.26 to 0.42), respectively (both *P* <0.001). On the other hand, use of the grading scale was associated with an increase in the overtriage rates (Table 3). Among the group of non-graded patients, there was a large difference in the undertriage rates between patients with no pre-hospital medical assessment and patients with pre-hospital medical management: 42% (95% CI 35% to 46%) versus 13% (95% CI 9% to 18%), respectively.

**Table 3:** **Univariate analysis according to whether patients were graded using an on-scene triage procedure (Graded group; n = 2,572 patients) or not (Non-graded group; n = 856 patients)**

**Variable**	**Graded group**	**Non graded group**	***P***
Age, mean year (SD)	37 (19)	37 (20)	0.73
Sex male, number (%)	1974 (77)	638 (75)	0.17
Mechanism of injury, number (%):			
Road traffic accidents	1127 (44)	368 (43)	0.03
Falls	472 (18)	180 (21)	
Skiing accidents	367 (14)	166 (20)	
Other mountain accidents	275 (11)	62 (7)	
Penetrating injuries	244 (9)	31 (4)	
Others	74 (3)	40 (5)	
Initial GCS, number (%):			
3 to 8	305 (12)	89 (11)	0.77
9 to 13	243 (9)	71 (9)	
14 to 15	2010 (79)	631 (80)	
Initial SBP <90 mmHg, number (%)	166 (6)	38 (5)	0.06
First assessment of SpO2 < 90%, number (%)	202 (8)	46 (6)	0.05
ISS ≥16, number (%)	1185 (47)	443 (52)	0.004
Pre-hospital medical assessment	2572 (100)	403 (48)	<0.001
Mortality, number (%)	176 (7)	31 (4)	0.001

GCS, Glasgow coma scale; ISS, injury severity score; SBP, systolic blood pressure; SD, standard deviation; SpO2, pulse oxygen saturation.

**Table 4:** **Undertriage and overtriage rates according to the definition used for appropriate triage**

**ACSCOT definition**							
	**Graded**		**Non-graded**				
	**number**	**% (95% CI)**	**number**	**% (95% CI)**	**ARR (95% CI)**	**RR (95% CI)**	***P***
Undertriage^a^	209	17.6 (15.4 ;19.8)	166	37.2 (32.7; 41.9)	−19.7 (−24.7; −14.7)	0.47 (0.40; 0.56)	<.001
Overtriage^b^	1047	76.6 (74.3; 78.8)	233	57.3 (52.3; 62.1)	+19.3 (14.0; 24.7)	1.34 (1.22; 1.46)	<.001
**TRENAU definition**							
	**Graded**		**Non-graded**				
	**number**	**% (95% CI)**	**number**	**% (95% CI)**	**ARR (95% CI)**	**RR (95% CI)**	***P***
Undertriage^c^	101	8.5 (7.0; 10.2)	115	25.8 (21.8; 30.1)	−17.3 (−21.7; 13.0)	0.33 (0.26; 0.42)	<.001
Overtriage^d^	804	58.8 (56.2; 61.4)	157	38.6 (33.8; 43.5)	+20.2 (14.8; 25.6)	1.52 (1.34; 1.74)	<.001

Definition of the American College of Surgeon’s Committee on Trauma (ACSCOT): ^a^undertriage = major trauma (ISS more than 15) admitted to trauma center level III; ^b^overtriage = not severe trauma (ISS less than 16) admitted to trauma center level I or II.

Definition of the Northern French Alps Trauma System (TRENAU): ^c^undertriage = major trauma (ISS more than 15) admitted initially to a level III trauma center before a transfer to a level I or II; or death in a trauma center level III; ^d^overtriage = not severe trauma (ISS less than 16) admitted to emergency room with an activation of trauma team in a level I or II trauma center. ARR: absolute risk reduction; CI: confidence interval; ISS, injury severity score; RR: relative risk.

The mortality rate at discharge from hospital was 6%, rising to 12% when ISS was greater than 15. Early mortality, as defined by death occurring within the first 48 hours after trauma, was due to traumatic brain injury (58%) or hemorrhagic shock (37%). Late mortality was attributed to brain death (32%), multiple organ failure (19%) or withdrawal of life-sustaining therapy (24%). The mortality rate predicted by the TRISS model was higher than the observed mortality, as shown by a W score of 2.3% (95% CI 1.9% to 2.7%) survivors in excess (*P* <0.01). Where adjusted for trauma severity, the standardized W score (Ws) remained significant: Ws = 2.0% (95% CI 1.4% to 2.6%; *P* <0.01).

## Discussion

Organized systems of trauma care are fundamental to achieving decreased mortality after severe trauma. The main objective of pre-hospital organization is allocating appropriate healthcare resources in accordance with the initial conditions of each trauma patient. The TRENAU combines together the American trauma system’s organization and the pre-hospital medical expertise available in France. In this study, we found that a grading system and a triage protocol could detect with accuracy the most severe trauma patients. The use of a pre-hospital triage procedure was associated with a significant reduction of the proportion of undertriaged patients at the expense of more overtriaged patients.

Our pre-hospital grading system was closely related to the ISS and intra-hospital mortality rate. Interestingly, the three classes of our grading protocol were similar to the three classes of the mechanism, Glasgow coma scale, age, and arterial pressure (MGAP) score developed by French EMS [15]. Our grading system included the French Vittel criteria and the response to treatment during the pre-hospital resuscitation. The grading at the scene is an easy-to-do process, appropriate to allow a quick medical decision after trauma and does not require the calculation of a score. The accuracy of the triage protocol according to the grading system to detect the most severe trauma patients showed relatively high sensitivity and low specificity. These results were consistent with the rates of overtriage and undertriage found in this study.

Undertriage is associated with a higher risk for unfavourable outcome while overtriage is a burden for hospital resources by monopolizing trauma care for patients without severe injuries [16]. Theoretically, the undertriage rate should not exceed 5% and overtriage should be maintained between 30% and 50% [11]. In our study, the undertriage rate in the Graded group was 18% or 9%, using the ACSCOT or TRENAU definition, respectively. This rate was comparable with undertriage rates found in other studies [3,17]. Surprisingly, an undertriage rate of 1% was found in Paris using a pre-hospital triage algorithm based on Vittel triage criteria recognition by emergency physicians [8]. This very low rate of undertriage could be due to the highly urban characteristics in that region. Interestingly, our undertriage rate went up to 37% and 26%, respectively, if no pre-hospital triage algorithm was used (Non-graded group). A significant proportion of our non-graded patients was not managed on the scene by an emergency physician. Interestingly, undertriage was particularly elevated in this subgroup of patients (42%) whereas non-graded patients managed by a physician had a lower rate of undertriage (13%). These findings suggest that a pre-hospital medical assessment of trauma severity enhances the quality of triage of trauma patients. In Norway, the triaging of patients by an on-scene physician was associated with a significant reduction of both undertriage and overtriage [18]. Conversely, the trauma triage protocol by paramedics in that country was associated with an undertriage rate of 17% and an overtriage rate of 66% [17]. In Japan, factors associated with an increased undertriage rate by paramedics were isolated moderate traumatic brain injury (odds ratio, OR = 9.10) and isolated pelvic trauma (OR = 14.2), both difficult to recognize in the field [19]. A direct medical assessment of victims by a trained physician could improve their initial triage by recognizing high-risk situations. An additional benefit to pre-hospital medical evaluation could be the pre-hospital use of Focused Assessment with Sonography for Trauma, in the early detection of the presence of blood in the abdomen [20]. Furthermore, the incorporation in the TRENAU grading system of responses to treatment during the pre-hospital resuscitation should help emergency physicians to grade trauma patients more appropriately. Collectively, these findings suggest that pre-hospital assessment by an emergency physician allows an early recognition of patients at risk for trauma-related complications and their allocation to the most suitable trauma center. These results also suggest that the TRENAU triage tool is more appropriate to a system involving pre-hospital physicians as compared to the ACSCOT triage tool for paramedics.

Undertriage is generally considered for the admission of severe trauma patients to a non-Level I trauma center, and vice versa for overtriage [11]. However, we also considered undertriage in situations where patients with an ISS >15 were admitted to a Level-III trauma center before secondarily being transferred to a Level-I or Level-II trauma center, or where patients with an ISS >15 died from their trauma related injuries in a Level-III trauma center. In the TRENAU system, we did, however, consider that patients with an ISS >15 could be treated adequately in a Level-III trauma center. For example, a severe trauma patient with an open leg fracture (limb AIS of 3) and an asymptomatic pneumothorax (chest AIS of 3) will have an ISS score of 18. This patient might have been admitted and successfully treated in a Level-III trauma center that corresponds to a general hospital near winter ski resorts, with the presence of both orthopedic and general surgeons. For undertriage assessment using the TRENAU definition, technical stops in a Level-III trauma center were not considered. Technical stop is a procedure in a Level-III trauma center near mountain sport resorts that permits a rapid extraction of trauma patients from an adverse environment, a minimal conditioning at the nearest hospital and a transfer to a Level-I or Level-II trauma center using the same emergency team. This TRENAU definition obviously led to reducing the undertriage rate, but might receive further external validation. These considerations for adequate triage explained the difference in sensitivity, specificity and rates of undertriage and overtriage found with the TRENAU versus the ACSCOT definitions.

Overtriage is also an important parameter to evaluate the performance of a trauma system. This indicator should be lower than 50% to avoid monopolization of trauma resources for non-severe patients [16]. In our study, the rate of overtriage and the specificity of the triage procedure were not optimal. However, the high overtriage and the low specificity found in this study might be explained by the recent creation of the trauma system leading to a more liberal orientation of patients suspected to have severe trauma to level-I or level-II trauma centers. The normalization of these indicators should be one goal of this trauma system in the coming years.

The mortality rate found in our cohort was relatively low (6%) compared to that reported in other European countries [21]. This may be due to a larger proportion of our trauma patients having an ISS score lower than 15 (52%). Interestingly, mortality was predominantly related to traumatic brain injury (58%), probably due to the high proportion of mountain sport-related accidents. The observed mortality rate was lower than predicted by the TRISS model. This result further supports our field triage scheme with a pre-hospital medical management and suggests a beneficial effect on mortality of this pre-hospital procedure, even in patients with an ISS lower than 15. This effect might be due to the pre-hospital medical management as well, because pre-hospital medical management was found to reduce mortality on day 30 after the insult (odds ratio 0.55, 95% confidence interval 0.32 to 0.94) in trauma patients [22]. However, our study was not designed to investigate a direct causal relationship between mortality and the implementation of the trauma system.

The present study has several limitations. First, the use of the grading system was not randomly assigned to patients in the Graded and Non-graded groups. Potential confounding factors due to the observational nature of the study might have altered comparisons between the two groups of patients. However, a randomized controlled trial to assess the impact of pre-hospital grading would have raised ethical issues. Second, the TRENAU was implemented in the French EMS system based on physician staffed-ambulances. The external validity of the pre-hospital grading system cannot be determined in the absence of on-scene medical evaluation. Our triage procedure appears effective at allocating severe trauma patients to the most suitable trauma center. It should be noted that the two groups of patients, that is, Graded and Non-graded, were evaluated during the same study period. Any changes in the management of trauma patients should be comparable between the two groups.

## Conclusions

A regional trauma system with a pre-hospital medical assessment and a triage procedure could detect with accuracy the most severe trauma patients and was associated with a reduction in the proportion of undertriaged patients. The triage procedure in a trauma system should include pre-hospital medical assessment using criteria based on the conditions of the trauma victims and their response to pre-hospital medical interventions.

## Key messages

- A regional trauma system with a pre-hospital triage procedure was associated with a reduction in the proportion of undertriaged patients.
- The triage procedure in a regional trauma system should include pre-hospital medical assessment
- Pre-hospital medical assessment is based on the initial conditions of the trauma victims and the response to medical interventions

## Additional file

Additional file 1:
**Northern French Alps Trauma System and French Vittel criteria.**

## Abbreviations

AIS: abbreviated injury score
ACSCOT: American College of Surgeons Committee on Trauma
CI: confidence interval
EMS: emergency medical service
GCS: Glasgow coma scale
ISS: injury severity score
OR: odds ratio
SD: standard deviation
SPO2: pulse oxygen saturation
TRENAU: Northern French Alps Trauma System
TRISS: trauma and injury severity score

## References

[1] Lendrum RA, Lockey DJ (2013). Trauma system development. Anaesthesia..

[2] MacKenzie EJ, Rivara FP, Jurkovich GJ, Nathens AB, Frey KP, Egleston BL (2006). A national evaluation of the effect of trauma-center care on mortality. N Engl J Med..

[3] van Laarhoven JJ, Lansink KW, van Heijl M, Lichtveld RA, Leenen LP (2014). Accuracy of the field triage protocol in selecting severely injured patients after high energy trauma. Injury..

[4] Mackersie RC (2006). Field triage, and the fragile supply of “optimal resources” for the care of the injured patient. Prehosp Emerg Care..

[5] Leppaniemi A (2005). Trauma systems in Europe. Curr Opin Crit Care..

[6] Bouzat P, Broux C, Ageron FX, Thony F, Arvieux C, Tonetti J (2013). Trauma network for severely injured patients. Ann Fr Anesth Reanim..

[7] Dick WF, Baskett PJ (1999). Recommendations for uniform reporting of data following major trauma–the Utstein style. A report of a working party of the International Trauma Anaesthesia and Critical Care Society (ITACCS). Resuscitation..

[8] Hamada SR, Gauss T, Duchateau FX, Truchot J, Harrois A, Raux M (2014). Evaluation of the performance of French physician-staffed emergency medical service in the triage of major trauma patients. J Trauma Acute Care Surg..

[9] Babaud J, Ridereau-Zins C, Bouhours G, Lebigot J, Le Gall R, Bertrais S (2012). Benefit of the Vittel criteria to determine the need for whole body scanning in a severe trauma patient. Diagn Interv Imaging..

[10] Sasser SM, Hunt RC, Faul M, Sugerman D, Pearson WS, Dulski T (2012). Guidelines for field triage of injured patients: recommendations of the National Expert Panel on Field Triage, 2011. MMWR Recomm Rep..

[11] Hospital and prehospital resources for optimal care of the injured patient. Committee on Trauma of the American College of Surgeons. Bull Am Coll Surg. 1986;71:4–23. 10278815

[12] Boyd CR, Tolson MA, Copes WS (1987). Evaluating trauma care: the TRISS method. Trauma Score and the Injury Severity Score. J Trauma..

[13] Champion HR, Sacco WJ, Copes WS (1995). Injury severity scoring again. J Trauma..

[14] Flora JD (1978). A method for comparing survival of burn patients to a standard survival curve. J Trauma..

[15] Sartorius D, Le Manach Y, David JS, Rancurel E, Smail N, Thicoipe M (2010). Mechanism, glasgow coma scale, age, and arterial pressure (MGAP): a new simple prehospital triage score to predict mortality in trauma patients. Crit Care Med..

[16] Newgard CD, Staudenmayer K, Hsia RY, Mann NC, Bulger EM, Holmes JF (2013). The cost of overtriage: more than one-third of low-risk injured patients were taken to major trauma centers. Health Aff (Millwood)..

[17] Rehn M, Eken T, Kruger AJ, Steen PA, Skaga NO, Lossius HM (2009). Precision of field triage in patients brought to a trauma centre after introducing trauma team activation guidelines. Scand J Trauma Resusc Emerg Med..

[18] Rehn M, Lossius HM, Tjosevik KE, Vetrhus M, Ostebo O, Eken T (2012). Efficacy of a two-tiered trauma team activation protocol in a Norwegian trauma centre. Br J Surg..

[19] Nakahara S, Matsuoka T, Ueno M, Mizushima Y, Ichikawa M, Yokota J (2010). Predictive factors for undertriage among severe blunt trauma patients: what enables them to slip through an established trauma triage protocol?. J Trauma..

[20] Tazarourte K, Dekadjevi H, Sapir D, Desmettre T, Libert N, Pasquier P (2010). Ultrasound and prehospital triage: a tool for limiting the undertriage. J Trauma..

[21] Huber-Wagner S, Lefering R, Qvick LM, Korner M, Kay MV, Pfeifer KJ (2009). Effect of whole-body CT during trauma resuscitation on survival: a retrospective, multicentre study. Lancet..

[22] Yeguiayan JM, Garrigue D, Binquet C, Jacquot C, Duranteau J, Martin C (2011). Medical pre-hospital management reduces mortality in severe blunt trauma: a prospective epidemiological study. Crit Care..

