# Supplementary material for: A regional trauma system to optimize the pre-hospital triage of trauma patients
Source: Crit Care. 2015 Mar 18;19(1):111. doi: 10.1186/s13054-015-0835-7 (PMC4403891; doi:10.1186/s13054-015-0835-7)
Supplement: Additional file 1: — Northern French Alps Trauma System and French Vittel criteria. [file 13054_2015_835_MOESM1_ESM.docx]

Additional file 1

Categorization of trauma centres in the French North Alpine Trauma network (TRENAU).

| Level | Available resources |
| --- | --- |
| Level I | 24/7: Emergency room, intensive care unit, all specialized surgeries, interventional radiology,  mass transfusion |
| Level II | 24/7: Emergency room, intensive care unit, general surgery,  conventional radiology with CT scan and interventional radiology, mass transfusion |
| Level IIII | 24/7: Emergency room and conventional radiology with CT scan |

Vittel Criteria

Step 1 (Physiological signs)

GCS < 13

SAP < 90 mmHg

SpO2 < 90%

Step 2 (Global assessment of speed and mechanism)

Ejection from vehicle

Death in same passenger compartment

Fail > 6 m

Victim thrown or projected

Global assessment of speed and potential injuries :

Vehicle deformation, estimated vehicle spped no helmet, no seat belt

Blast

Step 3 (Anatomical injuries)

Penetrating trauma of head, neck, thorax, abdomen, arms or legs

Flail chest

Severe burn

Pelvic fracture

Suspicion of spinal cord injury

Amputation at or above wrist or ankle level

Acute limb ischemia

Step 4 (resuscitation)

Mechanical ventilation

Intravascular filling > 1000 ml

Vasopressor
